# Revealing Dissociable Attention Biases in Chronic Smokers Through an Individual-Differences Approach

**DOI:** 10.1038/s41598-019-40957-0

**Published:** 2019-03-20

**Authors:** Chiara Della Libera, Thomas Zandonai, Lorenzo Zamboni, Elisa Santandrea, Marco Sandri, Fabio Lugoboni, Cristiano Chiamulera, Leonardo Chelazzi

**Affiliations:** 1 Department of Neurosciences, Biomedicine and Movement Sciences, University of Verona, Verona, Italy; 2 National Institute of Neuroscience, Verona, Italy; 3 Department of Diagnostics and Public Health, University of Verona, Verona, Italy; 4 Mind, Brain and Behavior Research Center CIMCYC, Department of Experimental Psychology, University of Granada, Granada, Spain; 5 Department of Internal Medicine, Unit of Addiction Medicine, Hospital Trust of Verona, Verona, Italy; 6 Data Methods and System Statistical Laboratory, Department of Economics and Management, University of Brescia, Brescia, Italy

## Abstract

Addiction is accompanied by attentional biases (AB), wherein drug-related cues grab attention independently of their perceptual salience. AB have emerged in different flavours depending on the experimental approach, and their clinical relevance is still debated. In chronic smokers we sought evidence for dissociable attention abnormalities that may play distinct roles in the clinical manifestations of the disorder. Fifty smokers performed a modified visual probe-task measuring two forms of AB and their temporal dynamics, and data on their personality traits and smoking history/status were collected. Two fully dissociable AB effects were found: A Global effect, reflecting the overall impact of smoke cues on attention, and a Location-specific effect, indexing the impact of smoke cues on visuospatial orienting. Importantly, the two effects could be neatly separated from one another as they: (i) unfolded with dissimilar temporal dynamics, (ii) were accounted for by different sets of predictors associated with personality traits and smoking history and (iii) were not correlated with one another. Importantly, the relevance of each of these two components in the single individual depends on a complex blend of personality traits and smoking habits, a result that future efforts addressing the clinical relevance of addiction-related AB should take into careful consideration.

## Introduction

Efficient behaviour in a busy world crucially depends on visual selective attention, so that limited processing resources can be devoted to task-relevant information while disregarding irrelevant and potentially distracting stimuli. Only objects that are selected by visual attention undergo in-depth processing, access awareness and guide overt behaviour^1,2^. Attentional priority, or the probability that a stimulus in the visual field will be selected at a given moment in time, has been traditionally described as depending on two classes of mechanisms^3^. On the one hand, *bottom-up* (or sensory-driven) mechanisms, reflecting low-level sensory-perceptual properties of the input, guide attention automatically towards conspicuous or unexpected stimuli; on the other, *top-down* (or goal-driven) mechanisms guide attention strategically towards task-relevant information^4^. However, growing evidence has recently challenged this dichotomous view, showing that attentional guidance is susceptible to other sources of control, generally attributable to the experience gained in the past with the same stimuli and context, sometimes known as selection history effects^5–8^. So, for instance, if the attentional selection of a given stimulus is extensively practiced^9–11^, or is systematically coupled with rewarding outcomes^12–14^, the attentional priority of the stimulus will be increased, irrespectively of its low-level properties (bottom-up information) and of current goals (top-down information).

Indeed, studies investigating cognitive abnormalities in addiction (e.g., nicotine addiction) have long shown that individuals who have developed a drug dependence tend to have their attention grabbed by stimuli in the environment that are associated with their substance of abuse^15–21^. Such *attentional biases* (AB) have been described in a large variety of addicted populations^22^ and are thought to result from the systematic coupling, which occurs as the addiction develops, between the perceptual and attentional processing of certain visual objects and the effects of the substance of abuse. Despite exerting different pharmacological effects, drugs of abuse share a similar ability to increase activation of dopaminergic neural circuits in the brain coding for the motivational relevance of sensory information, i.e., the reward system^23,24^. According to the *Incentive Sensitization Theory*^25,26^, the close contingency between the activation of the reward system during drug intake and the visual processing of the concurrent objects and context favours a transfer of the rewarding value from the drug itself to the associated visual stimuli and contexts. Following repeated experience, through associative learning, these initially neutral objects acquire an incentive value, becoming able to activate reward circuits on their own. The resulting increase of dopaminergic activity in structures such as the anterior cingulate gyrus, the amygdala and the nucleus accumbens, eventually leads to increased attentional priority of drug-related cues^27,28^. Interestingly, the same or highly similar mechanisms are thought to explain the biases of attention that emerge, in healthy individuals, following the delivery of (monetary) reward in association with certain visual stimuli^29,30^. In the latter studies, the perceptual and attentional processing of simple visual targets is followed by a reward signal, indicating the win of a variable amount of money. Critically, reward probability is biased a priori so that while responses to some stimuli lead more frequently to high rewards, responses to others lead instead to low rewards. As a result, highly rewarded stimuli acquire higher attentional priority and tend to be selected against any competing stimuli^6,31,32^. It is worth noting that under certain circumstances, and in certain populations, AB may also emerge as a tendency to avoid, rather than prioritize, motivationally relevant cues^33^, suggesting that more complex cognitive and motivational factors may further modulate the basic effects due to lower level stimulus-reward associations. Throughout this work we will refer to AB as to the more extensively and systematically studied phenomena which imply prioritization of drug-related cues.

Since their discovery, AB have been intensively studied in addiction research, because the burst of dopaminergic activity initiated by the detection of drug-related cues in the environment is thought to elicit increased levels of craving and eventually lead to drug-seeking behaviour, thus also favouring relapse in individuals who are trying to abstain^34,35^ (but see the work by Field and colleagues^36^ for a discussion of conflicting evidence). Interestingly, AB are not extinguished by common treatments of addiction, and, at least under certain circumstances, can still be observed in former addicts^16^.

Despite the widespread and ever-growing interest for AB within addiction research, several aspects of the phenomenon are still unclear, both at the theoretical and neurophysiological level^22,37^. For instance, one issue that is still unsolved is that the putative correlation between the AB measured in the individual subjects and their level of craving seems overall less robust than what should be expected^38^. This may be (at least partly) explained by the fact that the methods adopted to assess AB vary greatly across studies. AB are typically measured by means of behavioural paradigms borrowed from basic psychological research, and most of them assess either conflict monitoring and resolution processes^39^, or processes related to the deployment of visuospatial attention^16^. Importantly, these two kinds of processes are known to affect behaviour in distinct ways^40^ and to rely on largely independent neural networks^41^. Interestingly, although an AB can typically be revealed with both types of paradigm in addict populations, it appears that the two kinds of effect might index largely independent alterations of attention and reflect the working of independent processes^17^. In addition, even among studies adopting the same approach (i.e., either tapping conflict monitoring or visuospatial attention), differences in stimuli, task and experimental design make it sometimes difficult to generalize across studies by comparing directly, and especially numerically, their findings. An important implication of such methodological variability was recently put forward by a meta-analytic exercise, which attempted to assess the internal validity of AB measures, in the same way as it would be necessary for standardized diagnostic tests^42^. The results of this investigation were rather discouraging, with validity turning out to be quite low, and especially so for visuospatial attention tasks, highlighting that a sensible interpretation of AB effects always requires to account for the specific context in which they were measured, including the detailed task conditions as well as the features of the individuals taking part in the study.

Indeed, even within the same experimental setting, AB can be strongly influenced by individual differences^19,43–45^. For instance, in a previous study we found that AB for smoke cues was remarkably different in males and females (young University students)^45^. Not only did males show overall larger effects associated with smoke cues, but in all subjects the time-course of AB was further modulated by several individual traits: while AB emerging immediately after the onset of smoke cues was mainly associated with features of smoking behaviour (i.e., number of previous failed attempts to quit, number of cigarettes smoked in a day), the effects found after longer delays depended mainly on personality traits associated with general sensitivity to reward signals (i.e., scales assessing behavioural activation triggered by reward stimuli)^46^. So, it appeared that whether or not attention was initially seized by smoke cues depended on one’s severity of smoking status; however, whether it remained anchored to them for longer time periods was instead modulated by non-smoking related individual traits.

The evidence available so far strongly indicates that in order to grasp the true functional significance of addiction-related AB it is fundamental to explore cross-subject variability in its manifestations, and to capitalize on this variability both to understand the basic underlying mechanisms, and to appreciate their potential implications in clinical practice.

Here we set out to perform an exploratory investigation of these issues in a population of chronic smokers by means of a single task which was specifically designed to allow the assessment of two forms of AB triggered by smoke-related stimuli in the environment: one associated with the overall impact of smoke cues in the visual field (regardless of their specific location) as they compete with other stimuli for access to attentional selection; the other tied to the mechanisms mediating the shifting of visuospatial attention towards the location of smoke cues.

**Figure 1:**
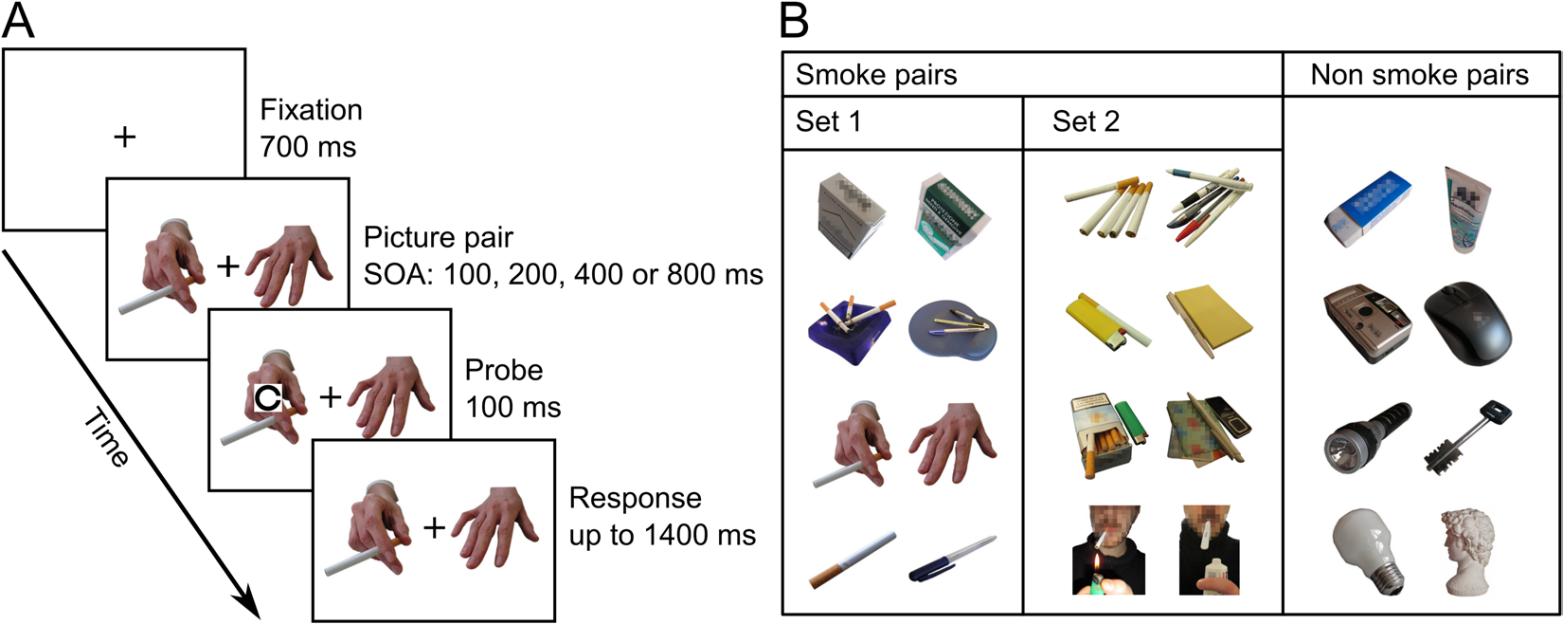
Experimental task and stimuli. **(A)** Sequence of events in a sample trial. **(B)** Smoke and non-smoke picture pairs used in the experiment. In order to avoid the possibility that task performance could be affected by incidental properties of the selected smoke-related images (i.e., non-specifically linked to their addiction-related content), two sets of smoke pairs were created and randomly assigned to each subject (Set 1 and Set 2 in the Figure). Preliminary analyses confirmed that the results obtained were not affected by the specific set of smoke-related pictures adopted in the given individual. Additionally, the specific exemplar of the given picture shown on the single trial was one of three variants, each portraying the relevant object from a different point of view (not shown). Photo credit: Nexus - Emergent Attention Lab (http://www.attention-lab.net/). Please note that the original pictures used in the experiment were modified in order to make all branded items as well as individual faces unrecognizable in the current publication.

Specifically, subjects took part in a behavioural experiment wherein, in each trial, two task irrelevant pictures appeared on the screen. In so-called *smoke trials* one of the pictures was smoke-related and the other was neutral, while on *non-smoke trials* both pictures were neutral (Fig. 1). After a variable interval (Stimulus Onset Asynchrony of 100, 200, 400 or 800 ms) the probe stimulus – to be discriminated – appeared on top of one of the two pictures. In smoke trials, half of the times the probe was placed on the smoke-related picture (*smoke match*), while on the other half it was placed on the neutral one (*non-smoke match*).

On the one hand, by comparing behavioural responses in smoke and non-smoke trials, we planned to assess whether the mere presence of smoke cues in the visual display, regardless of their specific location in relation to the probe, could impact performance, despite their being completely irrelevant for the task (*Global effect*). We reasoned that if smoke cues could automatically access selective attention and engage central processing, any concurrent activity might be delayed, possibly leading to behavioural costs in smoke trials. This situation could be similar to what is thought to occur in addiction Stroop tests^39^, where subjects are asked to name the ink colour of words printed in a coloured font, which can be either addiction-related or neutral. Typically costs are found in relation to addiction-related words, which are thought to derive from the need to ignore, and possibly suppress, the highly interfering semantic content of the word.

On the other hand, by zooming-in on smoke-trials, the comparison between responses to probes appearing on smoke vs. non-smoke pictures would allow us to explore more specifically the biasing of visuospatial attention towards (or away from) smoke-related cues (*Location-specific effect*). The unpredictably varying SOA further allowed to reveal the time-course of the effects of interest. In basic research on healthy individuals, this methodology enables the assessment of different effects associated with attentional deployment: at short SOAs target processing is facilitated at a location towards which attention has been initially drawn, while at longer SOAs (>200 ms) target processing at the same location is generally impaired, probably due to mechanisms reflecting the so-called Inhibition Of Return (IOR), which prevents visuospatial attention from returning to spatial positions that have been recently attended^47,48^.

Previous studies suggest that the mechanisms underlying Global and Location-specific forms of AB may rely on at least partly independent neural and cognitive mechanisms^40,41^; therefore, the two kinds of AB likely reflect, even within the same individual, the consequences of addiction-related brain plasticity affecting different neural networks^17^. Finally, by leveraging on a detailed profiling of our participants (Table 1), we aimed to further dissociate the two attentional alterations assessed in our task, by revealing their respective association with specific individual traits, including both smoke- and non-smoke related features; state-of-the-art correlational techniques were then applied to uncover the principal factors that are able to predict the occurrence of each form of AB at the single-subject level.

**Table 1:** Measures of individual differences considered in the study.

Individual profile information	Females (n = 21)	Males (n = 29)	Welch test for independent samples	
	Mean ± SE	Mean ± SE	t(df)	p-Value
Age	45.33 ± 2.27	47.93 ± 1.84	−0.89 (41.84)	0.38
Fagerström score	5.09 ± 0.44	5.62 ± 0.42	−0.86 (45.9)	0.39
QSU Brief score	27.76 ± 3.24	24.2 ± 2.33	0.89 (38.63)	0.37
Cigarettes per day	19.42 ± 1.8	22.41 ± 1.77	−1.18 (46.46)	0.24
Estimated COHb (%)	22.3 ± 3.1	28.7 ± 4.4	−1.17 (46.78)	0.24
Years of smoking	27.52 ± 2.18	31.86 ± 2.09	−1.43 (45.96)	0.16
Failed attempts to quit smoking	1.09 ± 0.3	1.17 ± 0.16	−0.22 (31.86)	0.82
STAI-Y State	1.56 ± 0.13	1.52 ± 0.08	0.21 (34.91)	0.83
STAI-Y Trait	1.91 ± 0.15	1.94 ± 0.08	−0.14 (31.52)	0.88
BAS Drive	0.61 ± 0.03	0.67 ± 0.03	−1.32 (42.98)	0.19
BAS Fun seeking	0.59 ± 0.05	0.60 ± 0.03	−0.28 (39.27)	0.78
BAS Reward responsiveness	0.77 ± 0.03	0.79 ± 0.03	−0.48 (46.94)	0.63
BIS	0.59 ± 0.02	0.61 ± 0.02	−0.63 (46.9)	0.51

Mean scores and standard error of the mean values (SE) are reported separately for male and female participants. No significant differences across groups emerged from Welch tests for independent samples.

## Statistical Analyses

The main focus of the statistical analyses was Reaction Time (RT) of correct responses at the computerized visual probe task. It is however worth noting that the results obtained with analyses of accuracy rates were in overall accord with those of RTs of correct responses, although they were less robust, likely due to the fact that the adopted speeded task emphasized RTs. Mean accuracy was 85% (±10% SD) across participants and conditions. Depending on the statistical procedures applied (see below), analyses were carried out on single-trial data or on performance means for each subject and condition. The Global effect and the Location-specific effect of smoke cues, as previously defined, were each initially investigated by means of a repeated measures ANOVA, exploring their overall occurrence in our participant sample. The ANOVA results were further corroborated by means of Linear Mixed-Effects models with random intercept, which – by considering single-trial data for each participant and task condition – should allow a more sensitive test for individual variability. Subsequently, separately for each SOA, we computed the Global effect of smoke cues (the related measure of AB) as the difference in RTs between smoke and non-smoke trials, and the Location-specific effect of smoke cues (the related measure of AB) as the difference, in smoke trials, between RTs to probes matching the location of the smoke cue vs. those to probes matching the location of the non-smoke cue in the pair. In both cases, positive values indicated a cost and negative values a facilitation associated with smoke-related information.

The effects computed for each subject were then submitted to a series of analytical procedures to reveal the possible link between each of the two effects and specific features (personality traits and smoking history) of the individual participant. These procedures followed two complementary approaches. On the one hand, Multiple Linear Regression (MLR) analyses^49^ were applied to reveal whether sources of cross-individual variability predicting Global and Location-specific effects of smoke cues at different SOAs were the same or different. Moreover, in the case of overlapping sets of relevant predictors, we could establish the differential impact of the predictors involved by analysing their relative weight^50^. On the other hand, generalized linear mixed-effects model trees (GLMM trees^51^) were employed to reveal whether overarching factors associated with individual differences could possibly account for the overall time-course of the two sorts of effect. GLMM trees use model-based recursive partitioning^52^ to detect subgroups showing significant differences in the interaction between the effect of interest (either Global or Location-specific effect) and SOA, and GLMMs to estimate the parameters of random-effects models in the identified subgroups.

In order to explore the relationships across the various predictors considered we additionally performed a Principal Components Analysis (PCA^53^) on the scores obtained from all subjects in our sample (see Supplement 1 for details). Four components were identified, each accounting for a different range of individual traits: Demographics (age, years of smoking, sex); Reward sensitivity (BAS subscales); Behavioural withdrawal (BIS scale, trait and state anxiety, number of failed attempts to quit smoking); Smoking behaviour (craving, dependence, number of cigarettes smoked in one day, estimated COHb).

Statistical analyses were performed in the statistical programming environment R^54^, with packages car^55^ and glmertree^56^, and with Stata 15^57^.

## Results

### Global effects of smoke cues

#### Overall performance

Mean RTs of correct responses were submitted to an ANOVA with Sex as a between-subjects factor, Trial type (smoke or non-smoke) and SOA (100, 200, 400 or 800 ms) as within-subjects factors. Trial type was marginally significant, *F*(1,48) = 3.76, *p* = 0.058, with responses to trials including a smoke-related image being slower than those with only neutral contents (Global effect: 610 ms vs. 601 ms). All other main effects and interactions were far from significance (all *p*s > 0.1). A linear mixed-effects model testing Trial type (smoke or non-smoke) and SOA (100, 200, 400 or 800 ms) as fixed effects and their interaction, with Subject as a random factor, revealed a significant effect of SOA, with performance at the shortest (SOA 100: 616.46 ms) being slower than at all the others (SOA 200 ms: 606.14 ms, *b* = −13.9, *p* = 0.011; SOA 400 ms: 594.54 ms, *b* = −22.01, *p* < 0.001; SOA 800 ms: 605.82 ms, *b* = −16.46, *p* = 0.003). All of the other effects or interactions were far from being significant (all *p*s > 0.1).

#### Individual differences

**Figure 2:**
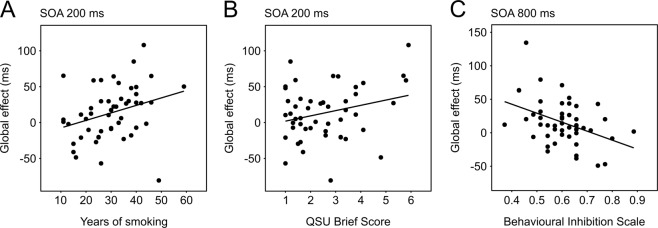
Main predictors of the Global effect of smoke cues as revealed by multiple linear regression analyses. The effect measured at SOA 200 ms was significantly predicted by Years of smoking **(A)** and by Craving, as assessed by QSU Brief score **(B)**. At SOA 800 ms the effect was significantly predicted by Behavioural Inhibition Score **(C)**.

The Global effects of smoke cues, computed separately for each SOA, were submitted to MLR models in which all sources of individual differences (Table 1) were initially considered as predictors; however following a preliminary test of multicollinearity within the factors, Age was excluded as its Variance Inflation Factor (VIF) was critically high (>4). In order to avoid overfitting, among the 13 initial factors we selected the best subset of predictors considering the results of two independent algorithms: a standard backward stepwise selection based on the AIC index and a factor selection following the incremental adjusted R-squared method^58^ *[note that it is generally suggested that in multiple linear regression models the ratio between the number of predictors and the number of independent observations should not be above 1/10. Given the 50 subjects in our sample we aimed at models including 5 predictors maximum]*. Explanatory models were considered if they included five or fewer predictors, and they reached a Multiple R-squared of at least 0.25 (explaining ≥ 25% of the variance; one statistically significant model was excluded due to this threshold, the results of which are reported in Supplement 2) (Table 2). Interestingly, two different models explained the Global effect of smoke cues at different time-points: the effect at SOA 200 ms was predicted by factors associated with smoking, namely Years of smoking, Craving (QSU Brief), Estimated COHb, Failed attempts to quit smoking and BAS Fun seeking (Multiple R-squared = 0.37, Adjusted R-squared = 0.30, *F*(5,44) = 5.26, *p* < 0.001; Fig. 2A,B). The effect at SOA 800 ms was instead predicted by factors mainly associated with personality: Behavioural inhibition (BIS), BAS Reward responsiveness, BAS Fun seeking, besides Failed attempts to quit smoking and Dependence (Fagerström) (Multiple R-squared = 0.33, Adjusted R-squared = 0.25, *F*(5,44) = 4.27, *p* = 0.003; Fig. 2C).

**Table 2:** Results of Multiple linear regressions on Global effect at SOA 200 ms and 800 ms, and on Location-specific effect at SOA 100 ms and 800 ms.

Modelled effect		Multiple Linear Regression parameters			
		Predictors	Estimated partial *β*	*p*-Value	Relative weight (%)
Global effect	SOA 200 ms	Years of smoking	1.78	0.0005	37.9
		QSU Brief	9.67	0.005	24.4
		Estimated COHb	−665.40	0.006	17.9
		BAS Fun seeking	−62.91	0.025	10
		Failed attempts to quit	5.09	0.218	9.8
	SOA 800 ms	BIS	−141.88	0.005	43.2
		Failed attempts to quit	6.89	0.093	27.1
		Fagerström	4.45	0.028	20.9
		BAS Reward responsiveness	75.40	0.075	5.4
		BAS Fun seeking	−54.74	0.138	3.4
Location-specific effect	SOA 100 ms	QSU Brief	19.35	0.0006	53.7
		BAS Reward responsiveness	102.02	0.019	21.2
		Fagerström	4.08	0.208	13.7
		STAI-Y State	−23.86	0.078	6.4
		Sex	15.80	0.241	4.9
	SOA 800 ms	Failed attempts to quit	13.14	0.012	27.5
		STAI-Y State	−26.48	0.021	24.9
		BAS Reward responsiveness	74.63	0.035	21.5
		Fagerström	−7.17	0.052	19.2
		Cigarettes per day	1.26	0.150	6.8

Each model comprises the best subset of predictors selected from the initial 13, ordered according to their weight, or the proportion of variance in the model explained by each predictor. *β* is the estimated partial regression coefficient for each predictor in the model and *p* value is the probability of its statistical significance.

In order to evaluate whether the Global effects observed at different SOAs would be related to one another within each participant, we carried out a series of linear correlation analyses, all leading to nonsignificant results (all *p*s > 0.1).

#### Individual differences and time-course analysis

**Figure 3:**
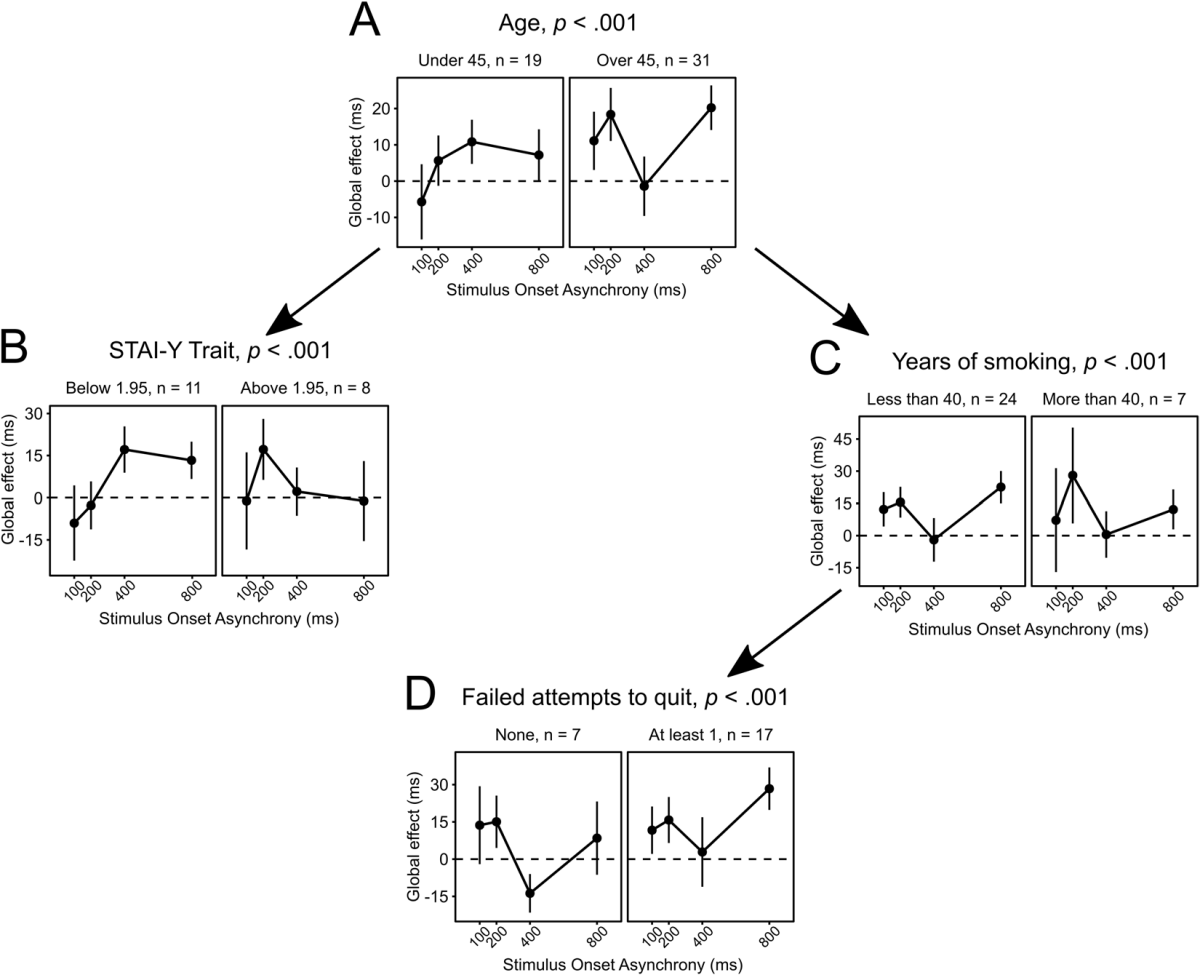
GLMM trees of our subject sample according to the time-course of the Global effect across different SOA durations. (**A**) The root node identified a significant partition in Age, below and above 45. (**B)** Younger subjects were then significantly partitioned in two groups according to their Trait Anxiety, below or above the 1.95 score at the STAI-Y Trait questionnaire. (**C)** Older subjects were instead significantly partitioned according to the length of their addition to smoking, with more or less than 40 Years of Smoking. (**D)** The subgroup of older subjects with less than 40 years of smoking was further significantly partitioned according to whether in the past they had tried to quit smoking or not.

Raw RT data were submitted to a model-based recursive partitioning (GLMM tree) algorithm exploring whether the interaction between Trial type and SOA – hence the full time-course of the Global effect – was systematically affected by individual characteristics. Several significant factors emerged (Fig. 3). The partitioning factor at the root node was Age, indicating that in younger participants the effect reversed, from benefit to cost, with increasing SOA (Fig. 3A, left panel). For older subjects, instead, the Global effect, when present, tended to be a cost at all timepoints (Fig. 3A, right panel). Younger participants were further partitioned according to Trait Anxiety, showing that the described trend was peculiar of subjects with lower anxiety (Fig. 3B, left panel). Anxious subjects, instead, barely showed any Global effect, and when present it consisted of a cost (Fig. 3B, right panel). Similarly, older subjects were subdivided according to Years of smoking. While in participants with a longer history of smoking the Global effect was an overall cost (Fig. 3C, right panel), subjects with fewer years of addiction were further subdivided according to Failed attempts to quit smoking. Interestingly, these two groups differed particularly at longer SOAs. Despite emerging immediately as a cost in both groups, with increasing SOA the Global effect was reduced (becoming a facilitation in some cases) in subjects who never tried to quit smoking in the past (Fig. 3D, left panel), while it kept increasing in subjects with previous failed attempts (Fig. 3D, right panel).

### Location-specific effects of smoke cues

#### Overall performance

Mean RTs of correct responses to smoke-trials were submitted to an ANOVA with Sex as a between-subjects factor, Probe location (smoke or non-smoke match) and SOA (100, 200, 400 or 800 ms) as within-subjects factors. None of the effects in the ANOVA were significant (all *p*s > 0.1). Overall, responses to probes appearing on the smoke-related image tended to be faster (609 ms vs 612 ms), but this effect was, on average, very small and far from being significant. These findings were replicated by the linear mixed-effects regression considering the fixed factors Probe location (smoke or non-smoke match), SOA (100, 200, 400 or 800 ms) and their interaction, with Subject as a random factor (all *p*s > 0.1).

#### Individual differences

**Figure 4:**
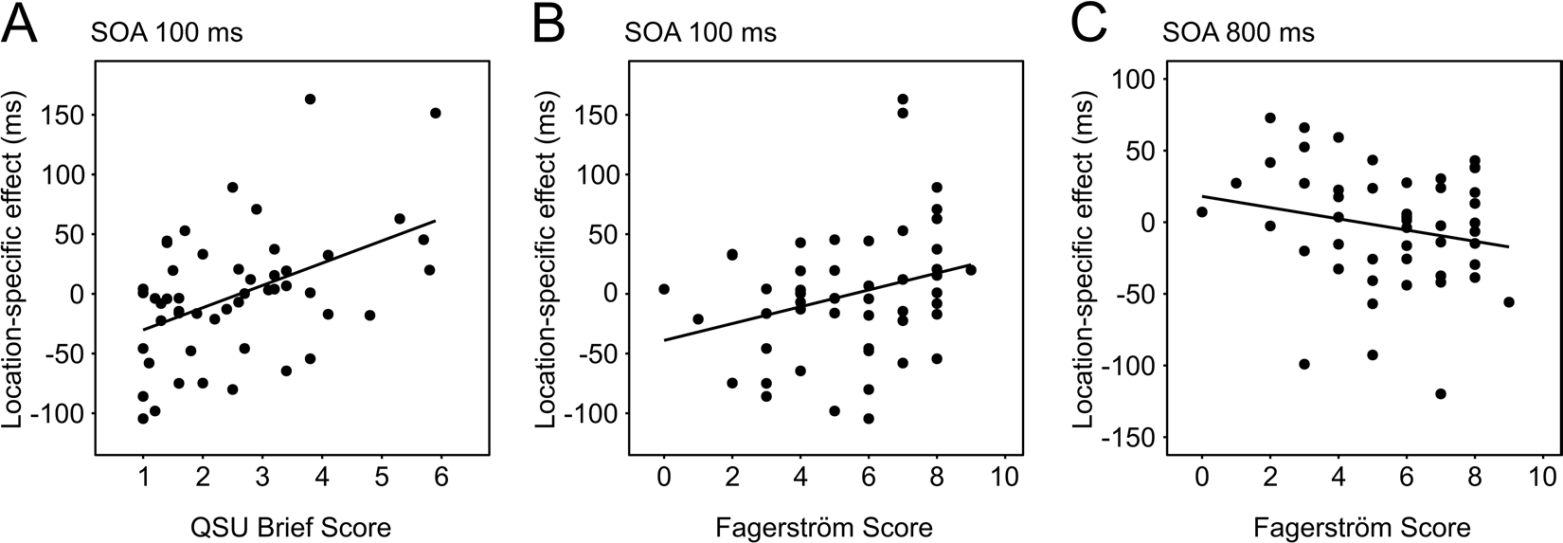
Main predictors of the Location-specific effect of smoke cues as revealed by multiple linear regression analyses. The effect measured at SOA 100 ms was significantly predicted by Craving, as assessed by QSU Brief score **(A)**, and the degree of Dependence, as assessed by the Fagerström score **(B)**. Fagerström score was also among the most influential predictors at SOA 800 ms **(C)**.

The rationale followed to select the best MLR models for the Location-specific effect was the same as described for the Global effect. Two significant models emerged (Table 2). The Location-specific effect of smoke cues at SOA 100 ms was predicted by a model comprising Craving (QSU Brief), BAS Reward responsiveness, Dependence (Fagerström), State Anxiety (STAI-Y State) and Sex (Multiple R-squared = 0.38, Adjusted R-squared = 0.31, *F*(5, 44) = 5.43, *p* = 0.0005; Fig. 4A). The same effect measured at SOA 800 ms was instead predicted by a model comprising Failed attempts to quit smoking, State Anxiety (STAI-Y State), BAS Reward responsiveness, Dependence (Fagerström) and Cigarettes per day (Multiple R-squared = 0.29, Adjusted R-squared = 0.21, *F*(5, 43) = 3.62, *p* = 0.008; Fig. 4B) *[one outlier was discarded from this analysis because the Bonferroni-corrected test of its Studentized residual was significant with p* < *0.05]*.

Interestingly, linear correlation analyses between the Location-specific effects at different SOAs revealed a mild anti-correlation between the effects measured at the shortest and longest intervals: effect at SOA 100 ms and 800 ms, Pearson’s *r* = −0.28, *t*(48) = −2.03, *p* = 0.047. All other tests were non-significant (all *p*s > 0.1).

#### Individual differences and time-course analysis

**Figure 5:**
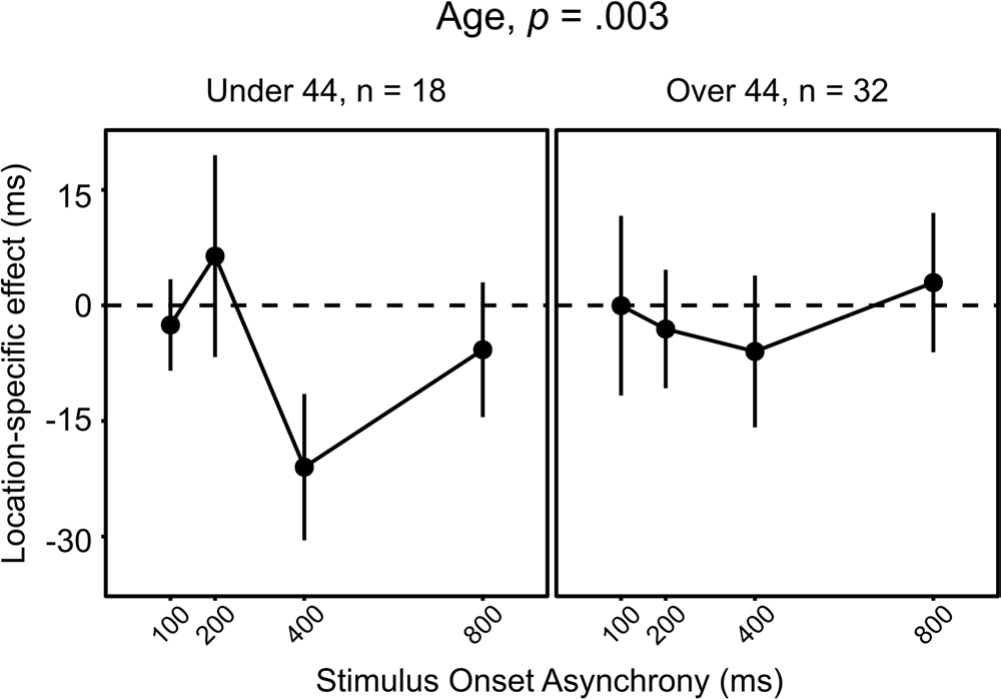
GLMM trees of our subject sample according to the time-course of the Location-specific effect across different SOA durations. The only significant partitioning factor identified was Age, with different models explaining the time-course of the effect in subjects under or over 40 years of age.

Following the same procedure described above, we explored the impact of individual differences on the time-course of the Location-specific effect by applying a GLMM tree algorithm. Here only Age had a significant impact on the relationship between SOA and Probe location (Fig. 5), indicating stronger time-based modulations of the effect in younger participants, in which target processing at the location of smoke-related stimuli was maximally facilitated at SOA 400 ms (Fig. 5, left panel).

### Global vs. Location-specific effects of smoke cues

Linear correlation tests were further performed to investigate the relationship between the Global and Location-specific effects of smoke cues assessed at the different SOAs. Importantly, all tests were far from being significant (all *p*s > 0.1). Hence, not only Global and Location-specific effects emerging at the same SOA in the same subject were not correlated, but also neither of the two effects could reliably predict the other at different points in time (see Supplement 3 for details).

## Discussion

To the best of our knowledge, the present study provides the first clear-cut evidence of dissociable forms of AB in nicotine addiction assessed in the same group of participants by means of a single behavioural task. Two forms of AB emerged, likely reflecting addiction-related plasticity in different attentional mechanisms, and they were distinctively and robustly associated with a variety of smoking- and personality-related individual differences. Moreover, the two forms of AB were characterized by distinct time courses within the single trial. Finally, the two effects were not correlated with one another.

The Global effect consisted of a cost in performance due to the presence of smoke cues in the visual display, reminiscent of the AB typically measured within conflict monitoring tasks, i.e., addiction Stroop tests^39^. In these tasks, stimuli are words printed in coloured font, and subjects must name the print colour while ignoring word meaning, typically showing slower responses if the test words are drug-related. Such cost suggests that while both neutral and drug-related words probably access semantic processing automatically^59^, drug-related words determine higher levels of interference, probably due to the triggering of resource-consuming drug-related thoughts and concerns that need to be suppressed, as well as greater engagement of attention^39^. Although similar competitive mechanisms likely underlie the Global effect found in our study, our data suggest that crucial information about the origin of this effect might be revealed by its time-course. We assessed the time-course of AB by randomly varying the time lapse from the onset of smoke cues to that of the task-relevant stimulus between 100, 200, 400 and 800 ms. As it is the case in a large variety of psychophysical experiments, the applied time manipulation establishes a context and the observations obtained should be always interpreted keeping that context in mind. While our observations therefore might provide an insight into the time-course of AB within this specific context (i.e., when SOAs vary randomly and equally between 100, 200, 400 and 800 ms), it is possible that the described phenomena are not anchored to the “absolute” timing of the underlying neural mechanisms, but rather to the relative probability of events within the session.

In our study, smoke cues trigger quickly, within 200 ms, a strong Global cost in subjects with a longer history of smoking and higher levels of craving (Table 2; Fig. 2A,B). Such slowing of responses is found in all trials with a smoke-related content, irrespectively of probe location, and we propose that it likely indexes interference with the ongoing task driven by the tendency of smoke-related information to rapidly attract attentional resources at the expenses of the primary task. However, when enough time is allowed before probe onset (i.e., 800 ms), this cost can be effectively reduced in subjects with higher inhibitory control (Table 2; Fig. 2C). On the other hand, subjects with low behavioural inhibition appear to be unable to overcome such interference, and might therefore represent a population with a higher risk of cue-induced peaks in craving, leading to possible relapse^35,37,60^.

The Location-specific effect instead might be akin to the AB commonly assessed by means of visual probe tasks^17^, which highlight the altered processing of targets that share the spatial location with the smoke cue. By varying SOA duration unpredictably across trials, we explored systematically the time-course of attentional prioritization of smoke cues^45^, revealing a Location-specific effect of smoke cues as early as 100 ms after their onset, which was particularly sensitive to individual differences. Both craving and the degree of dependence were among the most influential modulators of this effect: only subjects with lower craving and/or lower dependence expressed the expected benefit in performance associated with probes at the location of the smoke cue in the display. Participants reporting higher levels of craving, or with higher degrees of nicotine dependence tended instead to show an opposite trend, responding more slowly when probes appeared on smoke cues. This peculiar effect might result from our task design. Differently from other visual probe tasks, here task-irrelevant pictures remained visible throughout the whole trial, until response, with probes briefly appearing on top of one of them^45^. Previous studies^61,62^ have shown that in cases such as these, with cues capable of engaging attention very powerfully, the need to disengage from them in order to select a simultaneously displayed task-relevant stimulus may give rise to behavioural costs. Paradoxically, these costs may be enhanced when the old and the new stimulus share the same spatial coordinates, probably because the inhibitory traces left in place by processes involved in attentional disengagement from the cue might harm the selection of other stimuli at the same location^61,62^. Higher craving, as well as a higher degree of dependence, appear to have prolonged the lingering of attention on the smoke cue, thus interfering more powerfully with the processing of the overlapping probe^63^. Alternatively, it is possible that our subjects, who were patients referring to a smoking cessation centre, might have tried to avoid systematically any smoke cues, resulting in attention being immediately diverted from these objects in the display. However, the extremely early onset of the effect seems to argue against this possibility^64^.

Interestingly, craving ceased to be a reliable predictor of Location-specific AB at longer SOAs. The degree of smoking dependence on the other hand was still among the key predictors, but its relationship with the effect was reversed. At the longest SOA (800 ms), heavily dependent subjects, who showed higher costs earlier in time, now tended to exhibit faster responses to probes shown at the location of smoke cues, suggesting that in these subjects attention remained anchored at this location for a prolonged period of time. On the other hand, by this time, the competition between smoke cue and probe, which initially interfered with task response, might have dissipated. Interestingly, the finding of a significant anti-correlation between the Location-specific effects at the earliest and latest SOA is in keeping with theories on reflexive orienting of visuospatial attention, according to which the same neural mechanisms responsible for triggering immediate benefits in target processing at a cued location also determine processing costs later in time^47^.

Interestingly, recent research has shown that even when SOAs are fixed and predictable Location-specific AB varies significantly across consecutive trials^33^. Moreover, the claim has been made that the measures obtained from such intraindividual variability could be more informative of an individual’s sensitivity to smoke cues than the AB averaged within a whole experimental session. The inclusion of such dynamic measures of AB in future studies will allow for an even more accurate analysis of its intrinsic time course.

Besides these focal measures of AB, the overall analyses of their time-courses provided an additional perspective on the degree of individual variability of both effects. The aim of model-based recursive partitioning approaches was that of unveiling a possible pattern across the effects measured at different SOAs that would allow to identify groups of subjects with different attentional profiles. The assumption underlying this approach was that the attentional effects revealed at different delays from stimulus onset might reflect – to some extent – the influence of possibly super-ordinate, overarching mechanisms, accounting for the overall time-course of attentional deployment. Focused correlation tests between the different AB measures led to non-significant results either between different effects (Global vs. Location-specific) at the same SOA, or within the same effect across SOAs, with the only exception being the significant inverse relationship between early and late Location-specific AB discussed above. Interestingly, however, age emerged as an influential modulator of the unfolding in time of both effects, in line with evidence suggesting that the time-course of attentional deployment varies along the lifespan^65,66^. Additionally, the unfolding of the Global effect was further influenced by other predictors, suggesting that also the impact of individual traits on attentional deployment may vary with age. While anxiety seemed to impact more strongly on the expression of the bias in younger subjects, for older individuals the effect was more sensitive to the length of their addiction and their history of failed attempts to quit smoking. Interestingly, focused analyses on our predictors (Supplement 1) had revealed that the latter factor was not associated with other smoke-related data, but was instead significantly linked to traits of behavioural withdrawal. Given the high degree of parcellation of the data sample across partitions, these data should be interpreted with caution, but in the future the possibility of applying this analytical approach to larger samples might provide highly informative results.

The results obtained in the present study extend considerably the findings of our previous investigation, which focused on Location-specific effects of smoke cues in a selected population of young smokers^45^. Unlike what we found in that study, here gender never emerged among the key predictors of either form of AB, while craving (QSU Brief score) was highly determinant. Indeed, here the sample of participants was wider and much more diverse, so that the possible role of gender might have been obscured by other, more powerful sources of individual variability. Additionally, any group differences due to factors associated with sex hormones might have been less obvious because many women in the current sample were in the menopausal or peri-menopausal age range. On the other hand, the fact that all subjects in this sample had the purpose of quitting smoking might have emphasized the role of craving (QSU Brief), which systematically emerged as a robust modulator of the earliest manifestations of AB.

Overall, these results suggest that besides establishing that in addiction drug cues are subject to prioritized attentional processing, it is of fundamental importance for both basic and clinical research to understand the mechanisms that are subject to such addiction-related plasticity, in order to correctly evaluate their impact on other cognitive functions that eventually affect behaviour and decision making. It is possible in fact that the knowledge acquired on the basis of AB in addiction might have useful clinical applications, for instance predicting the risk of relapse^35^. Nevertheless, there are still several limitations that must be overcome in order to reach this goal, especially because in clinical and translational fields the label AB has grown to include virtually any form of altered processing found in association with drug cues, independently of the methods and tasks adopted for assessment. While such inclusiveness might be useful to construct a comprehensive framework of addiction-related cognitive biases, it might instead be harmful when the aim is that of reaching a deeper comprehension of the mechanisms involved in the establishment of incentive salience as well as in its reflection on attention and behaviour. Our findings suggest that AB assessed with different approaches provide independent, perhaps complementary, evidence of addiction-related attentional abnormalities. Importantly, within the same task the presence of addiction-related visual stimuli resulted in remarkably independent effects on performance, reflecting a multifaceted impact of these stimuli on different attentional mechanisms. Our results also highlight that, when investigating processing biases that depend on selective attention, it is imperative to take into proper consideration the temporal dimension, to explore the unfolding over time of the effects of interest, which is unlikely to emerge as clearly when the timing of task events is predictable (i.e., when SOA is fixed)^48^.

To sum up, our study provides the first evidence of how, within the same task and in the same population of chronic smokers, different and independent forms of AB for smoke-related stimuli can be isolated and characterized. Although both effects derive from an increased processing priority of smoke-related stimuli, they reflect the working of independent selective attention mechanisms which are supported by different neural networks and impact behaviour with a different time-course. Both forms of AB are distinctively influenced by individual differences, either associated with smoke-history or with general personality traits.

With these considerations in mind it seems clear that before establishing the general relevance of AB in the assessment and treatment of addiction-related cognitive disorders more effort is needed to understand and characterize the basic mechanisms underlying different types of AB and their expression in addicted populations with different traits and possibly different risk-levels. This can only be achieved through multidisciplinary approaches allowing a multi-level assessment of AB, in which individual differences are carefully taken into consideration. The results of the present study provide a first important step going in this direction.

## Methods and Materials

### Sample

The study was performed in accordance with the WMA Declaration of Helsinki regarding the ethical principles for research conducted on human subjects, and approved by the Ethics Committee for Clinical Trials, University Hospital Integrated Trust of Verona (protocol number: 292CESC). Participants were recruited among smokers seeking treatment at the Regional Smoking Cessation Centre in Verona. Prior to the start of the study, they read and signed an informed consent form and were screened with respect to the following exclusion criteria: ongoing therapy with neuroleptic drugs, depression (scoring higher than 60 at the Self-rating Depression Scale, SDS^67^), alcoholism (assessed through the Munich Alcoholism Test Scale 2, MALT2^68^), and assumption of a drug of abuse (heroin, cocaine, etc.) within the previous 12 months (self-report). Participants were also required to have normal or corrected-to-normal vision.

The sample size was determined through a priori estimations based on the findings of our previous study^45^, which consisted of a repeated-measures ANOVA with two within- and one between-subjects factor, carried out on the mean RTs in a behavioural task that was virtually identical to the one adopted here. Besides being necessary for preparing the experimental design, these analyses were also required for the study to be approved by the Ethics Committee for Clinical Trials. The standard deviation within the group was set to 80 ms, and the standard deviation explained by the effect was assumed to be 15 ms, which corresponded to a minimum partial *η*^2^ (ratio of variance explained) of 0.034 and a minimum clinically important effect size f of 0.19 (a small/medium effect size^69^). Setting the type I error at α = 0.05, the power 1 − β at 0.80, and the correlation between repeated measures at 0.1 (i.e., given the unknown correlation structure of repeated measures, we assumed a weak correlation as a precautionary measure), the minimum required total sample size was 50 subjects.

Considering a 10% rate of potential dropouts, the required total sample size was increased to 56 subjects. Consequently, fifty-six chronic smokers were initially recruited, but six of them were discarded because of poor task performance (at, or non-significantly different from chance). The final sample comprised fifty subjects (see Table 1 for details).

### Individual Profile Measures

Participants were administered a carbon monoxide (CO-) breath test (Smokerlyzer® instrument, Bedfont, England) and subsequently asked to fill-in several questionnaires. The data collected allowed us to build a multi-level profile for each subject comprising age, sex, smoking-related and personality-related information (Table 1). Smoking-related data consisted of both stable traits, such as the average number of smoked cigarettes per day, the number of failed attempts to quit smoking, the duration of smoking addiction in years, the degree of dependence on nicotine (as formally assessed by the Fagerström Questionnaire for Nicotine Dependence^70^), and of transient measures of the individuals’ smoking status, such as the level of craving for nicotine (as formally assessed by the Questionnaire of Smoking Urges – QSU Brief^71^) and an estimation of the concentration of blood carboxihemoglobin (COHb%), as indexed through the breath test. Personality-related data comprised the assessment of traits associated with sensitivity to reward signals and motivational drive (Behavioural Inhibition/Behavioural Activation Scales, BIS/BAS^46^) as well as anxiety (State-Trait Anxiety Inventory for Adults, STAI-Y^72^), which are domains of personality for which strong associations have been observed with smoking and addiction in general^45,73,74^.

### Experimental Task

After the initial assessment, subjects were introduced to the computerized behavioural task, which was programmed and run using E-Prime^75^. Both the experimental task and the stimulus set adopted in this study were developed in our laboratory and adapted from our previous work^45^. Participants sat at a computer desk in a quiet room while stimulus displays were shown on a 17-inch CRT monitor at 70 cm (Fig. 1A).

At the beginning of each trial a central fixation point appeared on the screen for 700 ms and was immediately followed by the onset of two pictures (4.7° × 4.7° of visual angle), one on the right and the other on the left, centred 3° away from fixation along the horizontal meridian (Fig. 1A). These pictures were selected from a set of sixteen that could be either smoking-related (e.g., a lighter) or neutral (e.g., stationery objects) and were never relevant for the task. After a given Stimulus Onset Asynchrony (SOA, 100, 200, 400 or 800 ms), randomly varying across trials, a small Landolt C of 1° in diameter was briefly shown on top of one of the two pictures, presenting the gap on the right or on the left side of its contour. This item was the target stimulus (probe) in the experimental task, and subjects were instructed to discriminate the right or left location of the gap by pressing as fast and accurately as possible one of two keys on the keyboard with their right-hand index or middle finger (key 1 for left and key 2 for right).

The pairs of pictures shown in the individual trial were chosen to be as similar as possible in overall shape and colour. Four pairs comprised one smoke-related and one neutral picture (smoke pairs); the remaining four pairs comprised two neutral pictures (non-smoke pairs) (Fig. 1B).

After a short practice, subjects performed 256 trials, half of which containing a smoke pair (smoke trials) and the other half a neutral one (non-smoke trials), randomly interleaved. The probe could appear equally often on the left or right of fixation, and required a left or right keypress with the same probability. Every picture in each pair was equally likely to be at the same location of the probe; therefore in smoke trials half of the times the probe was placed on the smoke-related picture (smoke match), while on the other half it was placed on the neutral one (non-smoke match).

## Supplementary information

Supplementary information

## Data Availability

The datasets generated and analysed during the current study are available from the corresponding author on reasonable request.
