# Supplementary material for: Revealing Dissociable Attention Biases in Chronic Smokers Through an Individual-Differences Approach
Source: Sci Rep. 2019 Mar 20;9:4930. doi: 10.1038/s41598-019-40957-0 (PMC6427017; doi:10.1038/s41598-019-40957-0)
Supplement: Supplementary file 1 — Supplementary information [file 41598_2019_40957_MOESM1_ESM.docx]

**Revealing Dissociable Attention Biases in Chronic Smokers Through an Individual-Differences Approach**

Chiara Della Libera1,2, Thomas Zandonai3,4, Lorenzo Zamboni5, Elisa Santandrea1,
Marco Sandri6, Fabio Lugoboni5, Cristiano Chiamulera2,3*, Leonardo Chelazzi1,2*

1. Department of Neurosciences, Biomedicine and Movement Sciences, University of Verona, Verona, Italy
2. National Institute of Neuroscience, Verona, Italy
3. Department of Diagnostics and Public Health, University of Verona, Verona, Italy
4. Mind, Brain and Behavior Research Center CIMCYC, Department of Experimental Psychology, University of Granada, Granada, Spain
5. Department of Internal Medicine, Unit of Addiction Medicine, Hospital Trust of Verona, Verona, Italy
6. Data Methods and System Statistical Laboratory, Department of Economics and Management, University of Brescia, Brescia, Italy

* These authors contributed equally to this work.

**CORRESPONDING AUTHOR:**

Leonardo Chelazzi, MD, PhD

Department of Neurosciences, Biomedicine and Movement Sciences

Section of Physiology and Psychology, University of Verona – Medical School,

Strada Le Grazie 8, 37134 Verona, Italy

Telephone: +39-045-8027149

Email: [leonardo.chelazzi@univr.it](mailto:leonardo.chelazzi@univr.it)

**SUPPLEMENTAL MATERIALS**

**Supplement 1**

***Principal Component Analysis of individual difference predictors***

In order to understand the underlying relationships among the variables considered as predictors, we performed a Principal Component Analysis (Jackson 1991) with an orthogonal varimax rotation procedure considering all the factors associated with individual differences (Table 1). An Eigenvalue of > 1 was used as a reference to define the number of Principal Components to be extracted from our data, leading to the identification of 4 components which accounted for very similar proportions of variance in the data collected through the computerized task. The first component, explaining 27% of variance, mainly consisted of demographic factors: Age, Years of smoking and a marginal contribution of Sex. The second, explaining 26% of variance, comprised measures of reward sensitivity, namely the three BAS subscales of the BIS/BAS questionnaire: BAS Drive, BAS fun seeking and BAS reward responsiveness. The third component explained 24% of the total variance, and comprised measures of behavioral withdrawal: the BIS scale of the BIS/BAS questionnaire, Trait and State Anxiety (measured with the STAI-Y questionnaire), and the number of failed attempts to quit smoking. The fourth component, explaining 23% of the total variance, comprised instead variables strictly associated with smoking behavior: Craving (measured through the QSU-Brief scale), Dependence (measured through the Fagerström scale), the average number of cigarettes smoked in one day and the estimated COHb.

**Table**

Loadings associated with each predictor in each principal component (rotation procedure)

| Principal component | Predictor | RPC 1 | RPC 2 | RPC 3 | RPC 4 |
| --- | --- | --- | --- | --- | --- |
| 1) Demographic data | Age | **0.92** | 0.02 | 0.08 | 0.01 |
|  | Years of smoking | **0.86** | 0.22 | 0.15 | 0.10 |
|  | Sex | **0.28** | 0.15 | -0.06 | 0.12 |
| 2) Reward sensitivity | BAS Reward responsiveness | 0.04 | **0.90** | -0.07 | -0.07 |
|  | BAS Drive | 0.00 | **0.79** | -0.05 | -0.02 |
|  | BAS Fun seeking | 0.27 | **0.77** | 0.12 | 0.09 |
| 3) Behavioural control | Trait Anxiety (STAI-Y Trait) | -0.08 | 0.29 | **0.81** | 0.04 |
|  | Failed attempts to quit smoking | 0.19 | -0.13 | **0.76** | -0.04 |
|  | State Anxiety (STAI-Y State) | -0.45 | 0.16 | **0.69** | 0.16 |
|  | Behavioural inhibition (BIS) | -0.11 | 0.22 | **-0.61** | 0.02 |
| 4) Smoking related data | Dependence (Fagerström) | 0.27 | -0.04 | 0.09 | **0.85** |
|  | Cigarettes per day | 0.11 | -0.03 | -0.03 | **0.85** |
|  | Craving (QSU Brief) | -0.35 | 0.20 | 0.10 | **0.59** |
|  | Estimated COHb | 0.48 | -0.17 | -0.12 | **0.51** |

**Supplement 2**

Here we report the results of a Multiple Linear Regression model which was not considered in the manuscript because it failed to reach the threshold of 25% explained total variance.

***Global effect at SOA 400 ms***: Two significant outliers were detected and removed from the analysis. The significant model included four predictors: State Anxiety (STAI-Y State), Cigarettes per day, Failed attempts to quit and Estimated COHb (Multiple R-squared = 0.21, Adjusted R-squared = 0.14, *F*(4,43) = 2.97, *p* = .03).

|  |  | Multiple Linear Regression parameters | | | |
| --- | --- | --- | --- | --- | --- |
| Modelled effect |  | Predictors | Estimated partial *β* | *p*-Value | Relative weight (%) |
| Global effect | SOA 400 ms | Failed attempts to quit | 8.90 | .065 | 41.5 |
|  |  | STAI-Y State | 14.28 | .086 | 32.6 |
|  |  | Estimated COHb | 342.54 | .105 | 16.7 |
|  |  | Cigarettes per day | -0.61 | .185 | 9.13 |

**Supplement 3**

Relationship between the measures of AB assessed at each SOA. None of the correlation analyses yielded significant results, suggesting that the two types of AB, Global and Location-specific, reflected at least partly independent cognitive processes. **(A)** SOA 100 ms: Pearson’s *r* = 0.111, *t*(48) = 0.781, *p* = .439. **(B)** SOA 200 ms: Pearson’s *r* = -0.092, *t*(48) = -0.645, *p* = .522. **(C)** SOA 400 ms: Pearson’s *r* = -0.204, *t*(48) = -1.449, *p* = .154. **(D)** SOA 800 ms: Pearson’s *r* = 0.119, *t*(48) = 0.832, *p* = .409.


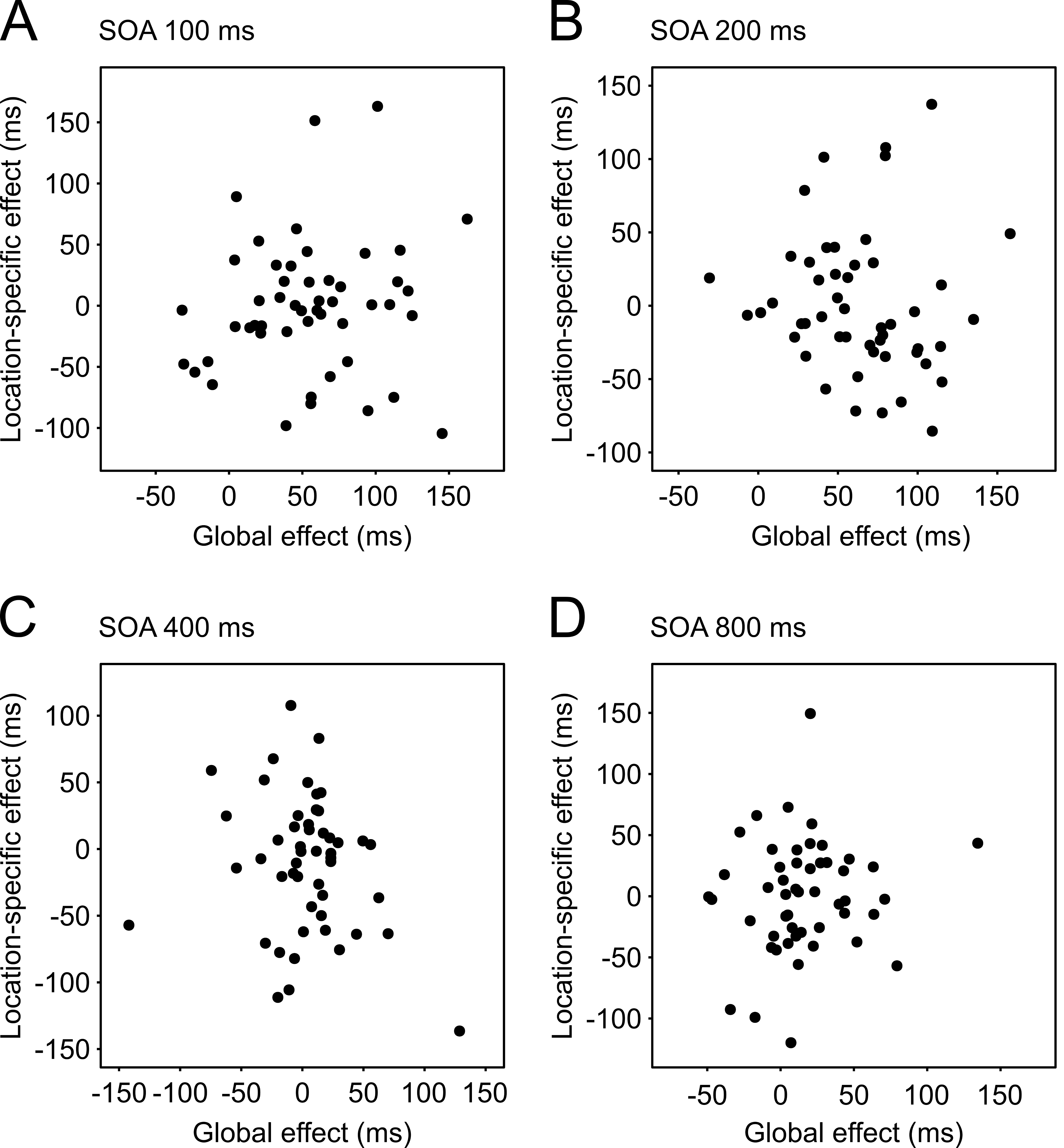


The relationship between Global and Location-specific effects of smoke cues was also investigated across SOAs. All of the statistical effects were far from being significant, suggesting that one type of AB at a given SOA was in no way predictive of the other, at a different point in time.

***Global effect at SOA 100 ms vs.:*** Location-specific effect at SOA 200 ms: Pearson’s *r* = -0.002, *t*(48) = -0.019, *p* = .984; Location-specific effect at SOA 400 ms: Pearson’s *r* = -0.158, *t*(48) = -1.115, *p* = .270; Location-specific effect at SOA 800 ms: Pearson’s *r* = -0.189, *t*(48) = -1.337, *p* = .187.

***Global effect at SOA 200 ms vs.:*** Location-specific effect at SOA 100 ms: Pearson’s *r* = 0.153, *t*(48) = 1.078, *p* = .286; Location-specific effect at SOA 400 ms: Pearson’s *r* = -0.075, *t*(48) = -0.521, *p* = .605; Location-specific effect at SOA 800 ms: Pearson’s *r* = -0.075, *t*(48) = -0.521, *p* = .604.

***Global effect at SOA 400 ms vs.:*** Location-specific effect at SOA 100 ms: Pearson’s *r* = -0.132, *t*(48) = -0.923, *p* = .359; Location-specific effect at SOA 200 ms: Pearson’s *r* = 0.135, *t*(48) = 0.947, *p* = .348; Location-specific effect at SOA 800 ms: Pearson’s *r* = 0.091, *t*(48) = 0.091, *p* = .526.

***Global effect at SOA 800 ms vs.:*** Location-specific effect at SOA 100 ms: Pearson’s *r* = -0.092, *t*(48) = -0.643, *p* = .522; Location-specific effect at SOA 200 ms: Pearson’s *r* = 0.038, *t*(48) = 0.270, *p* = .788; Location-specific effect at SOA 400 ms: Pearson’s *r* = 0.196, *t*(48) = 1.391, *p* = .170.

***Location-specific effect at SOA 100 ms vs.:*** Global effect at SOA 200 ms: Pearson’s *r* = 0.153, *t*(48) = 1.078, *p* = .286; Global effect at SOA 400 ms: Pearson’s *r* = -0.132, *t*(48) = -0.924, *p* = .359; Global effect at SOA 800 ms: Pearson’s *r* = -0.092, *t*(48) = -0.643, *p* = .522.

***Location-specific effect at SOA 200 ms vs.:*** Global effect at SOA 100 ms: Pearson’s *r* = -0.002, *t*(48) = -0.019, *p* = .984; Global effect at SOA 400 ms: Pearson’s *r* = 0.135, *t*(48) = 0.947, *p* = .348; Global effect at SOA 800 ms: Pearson’s *r* = 0.038, *t*(48) = 0.270, *p* = .788.

***Location-specific effect at SOA 400 ms vs.:*** Global effect at SOA 100 ms: Pearson’s *r* = -0.158, *t*(48) = -1.115, *p* = .270; Global effect at SOA 200 ms: Pearson’s *r* = -0.075, *t*(48) = -0.521, *p* = .604; Global effect at SOA 800 ms: Pearson’s *r* = 0.196, *t*(48) = 1.391, *p* = .170.

***Location-specific effect at SOA 800 ms vs.:*** Global effect at SOA 100 ms: Pearson’s *r* = -0.189, *t*(48) = -1.337, *p* = .187; Global effect at SOA 200 ms: Pearson’s *r* = -0.075, *t*(48) = -0.521, *p* = .604; Global effect at SOA 400 ms: Pearson’s *r* = 0.091, *t*(48) = 0.638 *p* = .526.
